# Severe acute malnutrition and mortality in children in the community: Comparison of indicators in a multi-country pooled analysis

**DOI:** 10.1371/journal.pone.0219745

**Published:** 2019-08-06

**Authors:** Catherine Schwinger, Michael H. Golden, Emmanuel Grellety, Dominique Roberfroid, Benjamin Guesdon

**Affiliations:** 1 Centre for Intervention Science in Maternal and Child Health, Centre for International Health, University of Bergen, Bergen, Norway; 2 Department of Medicine and Therapeutics, University of Aberdeen, Aberdeen, Scotland, United Kingdom; 3 Research Center Health Policy and Systems - International Health, School of Public Health, Université Libre de Bruxelles, Brussels, Belgium; 4 Belgian Health Care Knowledge Centre (KCE), Brussels, Belgium; 5 Action Against Hunger, Paris, France; Institute of Economic Growth, INDIA

## Abstract

**Objectives:**

This study aims to describe the mortality risk of children in the community who had severe acute malnutrition (SAM) defined by either a mid-upper arm circumference (MUAC) <115mm, a low weight-for-height Z-score (WHZ) <-3 or both criteria.

**Methods:**

We pooled individual-level data from children aged 6–59 months enrolled in 3 community-based studies in the Democratic Republic of the Congo (DRC), Senegal and Nepal. We estimate the mortality hazard using Cox proportional hazard models in groups defined by either anthropometric indicator.

**Results:**

In total, we had 49,001 time points provided by 15,060 children available for analysis, summing to a total of 143,512 person-months. We found an increasing death rate with a deteriorating nutritional status for all anthropometrical indicators. Children identified as SAM only by a low MUAC (<115mm) and those identified only by a low WHZ (Z-score <-3) had a similar mortality hazard which was about 4 times higher than those without an anthropometric deficit. Having both a low MUAC and a low WHZ was associated with an 8 times higher hazard of dying compared to children within the normal range. The 2 indicators identified a different set of children; the proportion of children identified by both indicators independently ranged from 7% in the DRC cohort, to 35% and 37% in the Senegal and the Nepal cohort respectively.

**Conclusion:**

In the light of an increasing popularity of using MUAC as the sole indicator to identify SAM children, we show that children who have a low WHZ, but a MUAC above the cut-off would be omitted from diagnosis and treatment despite having a similar risk of death.

## Introduction

The term severe acute malnutrition (SAM) commonly refers to severely wasted children who require urgent admission to a therapeutic feeding program. Since 2009, WHO and UNICEF have recommended a mid-upper arm circumference (MUAC) <115 mm or a weight-for-height/length Z-score (WHZ) <-3, as well as nutritional edema, as independent criteria to define SAM in children over 6 months of age [1]. Because there are now two independent anthropometric criteria, malnourished children segregate into three separate categories: (1) only MUAC <115mm, (2) only WHZ <-3 and (3) both MUAC <115mm and WHZ<-3. Despite the fact that these are both measures of “thinness” they usually do not identify the same children. The diagnostic discrepancy between low MUAC and low WHZ diagnoses has been repeatedly confirmed since 2009, by the analysis of cross-sectional surveys [2–5], and by the analysis of admissions to therapeutic feeding programs [6–8]. Recently, the analysis of more than 1,800 cross-sectional surveys’ datasets from 47 countries shed a new light on the magnitude of this discrepancy. It showed that only 16.5% of children fulfilled both defining criteria (MUAC <115mm and WHZ <-3) [9]. Both the magnitude and direction of the diagnostic discrepancies were highly variable across countries. Particularly in the Sahel and South-East Asia where most cases are located [10], it appears that a low WHZ contributes to a relatively high proportion of all SAM children. These children would remain undetected by a program using MUAC as only criterion for admission [9].

While the recommendation to use MUAC <115mm and WHZ <-3 independently was reiterated by WHO in 2013 [11], the use of absolute MUAC as the only measurement for case finding and admission to therapeutic feeding programs has been increasingly promoted and applied in recent years by public and private stakeholders involved in SAM management programs [12–15]. In spite of the need to simplify identification of children with SAM, the demand for more investigation into the clinical and physiological significance of this diagnostic discrepancy and other consequences of shifting to MUAC-only programming have been largely ignored [9, 11, 16, 17]. SAM management programs are primarily interested in reducing short-term death of SAM children, and therefore one of the critical elements in the debate around MUAC-only programming is the extent to which mortality risks differ between the defined categories. Recently, an empirical study pooling data from >70,000 children admitted for SAM treatment, as well as a meta-analysis using 21 datasets concluded that there was no difference in mortality risk between those children identified with a low MUAC and those with a low WHZ [18, 19]. However, only a limited number of these studies focused on mortality risk based on observations in the community in the absence of treatment and none of them compared the elevation of mortality risks associated with each diagnosis category. A recent pooled-analysis of existing community cohort studies has confirmed a dramatically elevated mortality risk in children with WHZ <-3 which was 11.6 times higher than in children without this anthropometric deficit [20]. No such analysis has been published for children with MUAC <115mm, and any mortality risk discrepancy between the two criteria has not been determined by direct comparison in the same community.

The objective of the present study was to describe the all-cause mortality hazard by categories of deficits in MUAC and/or WHZ. To inform the existing debate surrounding MUAC-only programming, we aimed to describe the relative mortality hazard of children in the community who would be identified under a MUAC-only or WHZ-only program (SAM by MUAC <115mm or WHZ <-3). In addition, we aimed to describe the mortality hazard of those children who would be excluded from treatment under those programs (i.e. children with single deficits of either only MUAC <115mm or only WHZ <-3 without concurrently having the other deficit), as well as those satisfying both criteria. We obtained rare longitudinal cohort datasets of children in the community not enrolled into nutritional programs, which had all the required anthropometric measurements and regular follow-up to determine survival. The effect of age, sex, and concomitant stunting on these relative mortality hazards were also examined, as well as the effect of anthropometric deficits on death due to specific causes.

## Methods

### Selection of studies

We identified previously completed large community-based prospective studies, which measured weight, length/height, MUAC and the vital status of participating children during follow-up, and contacted principal investigators to ask for their individual-level data. We obtained anonymous data for three studies; two prospective cohort studies that initially aimed to describe the links between anthropometric deficits and child mortality in Senegal [21] and Democratic Republic of the Congo (DRC) [22], and one trial from Nepal that investigated the effects of Vitamin A supplementation on child mortality and other outcomes [23]. Data collection took place in 1983–1986 in Senegal, 1989–1992 in DRC, and 1989–1990 in Nepal.

### Ethical statement

As we used anonymous published secondary data only, formal ethical clearance was not required.

### Study population and follow-up

For each original study, the only original eligibility criterion for study enrollment was an age between 0 and 5 years and residence within the catchment area of the study. For the DRC study, it is not known how many children in the study area declined participation. For the Senegal study and the Nepal study, 88.5% and 96% of eligible children in the community were enrolled respectively. In the Nepal study, height was only measured in a sub-set of the children, which was used for this analysis. It is not clear how the sub-set was selected. Children were included in the current analysis, if they had at least one visit during which their weight, length/height and MUAC were taken in the period where they were between 6 and 60 months of age, and a subsequent visit with their outcome recorded. Each eligible child contributed time to the study until s/he reached 60 months of age, died, was lost-to follow up, or was present at the administrative end of follow-up. For none of the studies were data available on whether the children did or did not have edema during their assessment.

### Definitions of outcome and determinant variables

For this study, the main determinant variables were absolute mid-upper-arm circumference (MUAC; in cm unadjusted for age, height or otherwise related to standards), and weight-for-height/length Z-scores (WHZ). The anthropometric indicators were each classified into 4 categories: (1) reference, (2) mild, (3) moderate and (4) severe deficits in accordance with WHO guidelines [24, 25]. The cut-off values are shown in Table 1. Children with a MUAC<115mm and/or <-3 Z-scores were defined as having severe acute malnutrition (SAM) and would have been eligible for inclusion in a therapeutic treatment program [11]. We categorized children as SAM by MUAC-only, WHZ-only and both MUAC and WHZ. Children in category MUAC-only and WHZ-only represent those who would be excluded from a program if only the opposite indictor is used. Assessing the mortality risk in the last category can show potential additive effects where both indicators are under the cut-off points for SAM.

**Table 1:** Category definition of the determinant variables mid-upper arm circumference (MUAC; in mm) and weight-for-height Z-score (WHZ).

Category	MUAC (mm)	WHZ (Z-score)
Reference	≥135	≥-1
Mild	≥125, <135	≥-2, <-1
Moderate	≥115, <125	≥-3, <-2
Severe	<115	<-3

The main outcome variable was mortality regardless of cause. The individual studies ascertained the vital status of participants at regular study visits and assigned causes of death using the verbal autopsy methods current at the time of the study. On average, children were visited every 3 months in the DRC study, every 6 months in the Senegal study and every 4 months in the Nepal study. In each study, the exact date of death was documented. In the cause-specific analyses, we classified deaths as those due to diarrheal diseases, acute respiratory infections, measles, malaria, trauma and unknown cause.

### Statistical analyses

Data were analyzed with Stata (version 15; StataCorp LP, College Station, Texas). We pooled the 3 datasets, and calculated all Z-scores according to WHO 2006 Child Growth Standards [26]. Because we categorized Z-scores, we did not delete extreme values, as it is very likely that an extreme value would be below -3 in reality and would therefore be correctly assigned to the severe category. However, only few values were <-7 for HAZ and WAZ, and <-6 for WHZ and could be checked manually. Three values for HAZ (0.01%), 13 values for WHZ (0.03%) and 6 values for WAZ (0.01%) were regarded as implausible and deleted together with their corresponding raw value(s) for height and/or weight.

We report case-fatality rates (CFR) defined as numbers of death per 100 child-months. We also report deaths per 10,000 children per day, which is the unit that is usually used when assessing death rates in humanitarian situations.

We estimated mortality hazard ratios for children in each exposure category defined at the start of each observation interval relative to the specified reference category. We used Cox proportional hazards regression models with robust sandwich covariance matrix estimates to account for repeated measurements for each child. Due to left truncation, we used child’s age (in months) as the time scale. Both the log-log plots and the Schoenfeld residuals indicated a violation of the assumption of the proportional hazards for the variable “cohort”, but not for either anthropometric variable or age. We therefore specified the cohort variable as a stratum in the main model (Stata command “strata”) to allow for separate baseline hazards [27]. Sex was initially included in each model, but was not significant at a 0.05 level and therefore removed. We tested for interactions for the variables cohort, age and height-for-age Z-score. We included interaction terms for each of these 3 variables separately in all the bivariable models with the different anthropometric indicators. There was a significant interaction (p <0.05) between the anthropometric indices and both study cohort and age; therefore, we report additional stratified analyses. Hazard ratios with a 95% confidence interval (CI) that does not cross 1.0 and p-values <0.05 were regarded as statistically significant. For the categories of SAM by MUAC and/or WHZ, we defined 2 reference categories. The first uses those that are non-SAM with a MUAC ≥ 115mm and WHZ ≥-3 as a reference to represent those that would not be included in a nutritional program. The second uses those defined as of normal anthropometric status, i.e. a MUAC ≥135mm and a WHZ ≥-1, as reference to make a comparison with non-malnourished children.

In order to examine the potential for measurement error to affect the results, an additional analysis was undertaken. We defined a measurement as implausible based on our long-standing clinical experience and deleted the raw values and associated Z-scores in the two adjacent measurements, if a child lost height (964 (2%) occurrences) or gained >4 cm within 3 months (1,335 (2.7%) occurrences), lost or gained >20% of its’ weight (409 (0.8%) and 36 (0.07%) occurrences) or lost or gained more than 2 cm in MUAC within 3 months (153 and 168 occurrences (both 0.3%)). Analyses with these additional cleaning criteria resulted in very similar regression coefficients; the results using these latter criteria are given in the supporting information S1 Table.

## Results

There were 17,520 children in the three original datasets. Of the 68,888 measurements, 6,151 measurements were done when the child was younger than 6 months, 7,995 when the child was older than 59 months, and at 10 time points the age of the child was missing; thus, these data were excluded from this analysis. At 5,731 time points either WHZ and/or MUAC were not recorded, leaving 49,001 time points provided by 15,060 children for analysis. This resulted in a total of 143,512 person-months of observation available for analysis. Of the eligible children, 49% were female. There were 749 deaths recorded (5.0% of the study population) for which children had records of their anthropometric indicators at the start of the measurement period. Mortality was higher in the Senegal dataset (10.4%) than in the two other datasets. Of the children that died, 48% were female and the median age at death was 26 months (interquartile range 18–35 months). The mean WHZ (SD) was -0.22 (1.03) in the DRC cohort, -0.86 (1.03) in the Senegal cohort, and -0.41 (1.15) in the Nepal cohort. The children in Nepal had the highest prevalence of stunting and wasting based on HAZ and WHZ; those in DRC had a higher percent with a low absolute MUAC and MUAC-for-age Z-score. Table 2 shows the main cohort characteristics.

**Table 2:** Cohort characteristics and anthropometric status for all children aged 6–59 months in the pooled analysis and for each original study separately.

	Total	DRC	Senegal	Nepal
Number of children, n	15,060	4,585	5,143	5,332
Mean person-time per person, mo (SD)	10.1 (6.2)	9.6 (5.3)	9.7 (7.0)	11.1 (5.8)
Mean measurement interval, mo (SD)	4.1 (1.6)	3.0 (0.8)	6.2 (2.0)	4.1 (0.7)
Deaths, n (%)	749 (5.0%)	128 (2.8%)	537 (10.4%)	84 (1.6%)
Female, %	48.8	48.5	49.5	48.6
Age <24 mo, %	34.1	33.2	37.1	32.9
Stunted (HAZ <-2), %	58.2	65.1	31.0	70.1
Wasted (WHZ <-2), %	8.5	4.6	8.2	12.6
Low MUAC-for-age Z-score (<-2), %	31.8	50.4	14.7	25.3
Low MUAC (<125 mm), %	17.5	28.8	8.7	12.6

**Fig 1:**
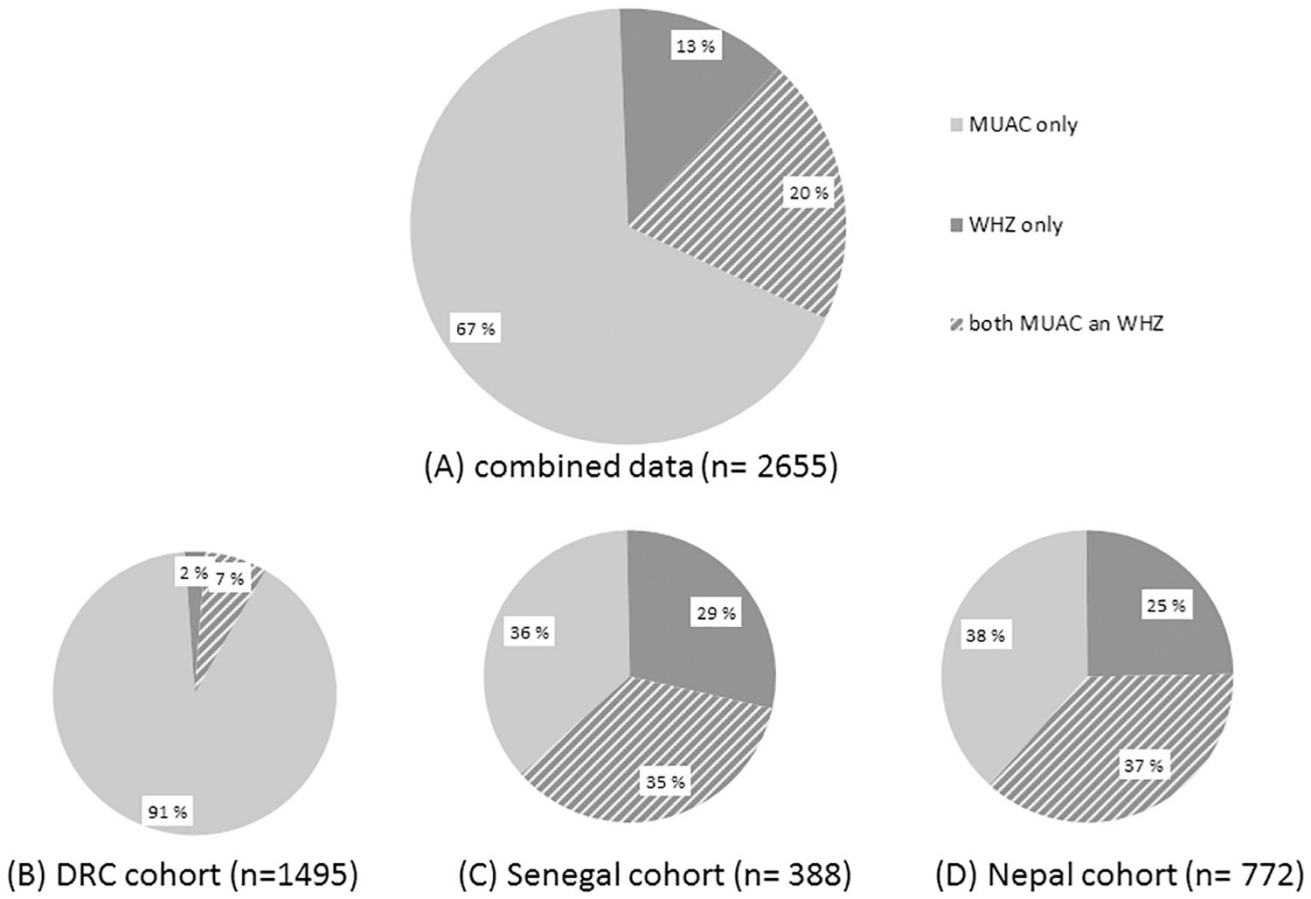
Proportion of children defined as SAM by MUAC-only (solid light grey area), WHZ-only (solid dark grey area) and both MUAC and WHZ (striped area) for (A) the combined data, (B) the DRC cohort, (C) the Senegal cohort, and (D) the Nepal cohort.

Of all measurements points with data available for MUAC and WHZ, 2,317 (4.7%) were <115mm for MUAC, 865 (1.8%) were <-3 Z-scores for WHZ, and 527 (1.1%) were under the cut-off points for both MUAC and WHZ. Of all measurements categorized as SAM, 338 measurements (13%) were under the cut-off for WHZ only and thus would be excluded from SAM diagnosis using MUAC as sole indicator (Fig 1A). Similarly, 1,790 measurements (67%) were below the cut-off for MUAC-only and these would be excluded if only WHZ was used for diagnosis. However, these proportions differ in the 3 cohorts, in particular in the DRC cohort compared to the Senegal and Nepal cohorts (Fig 1B–1D).

**Fig 2:**
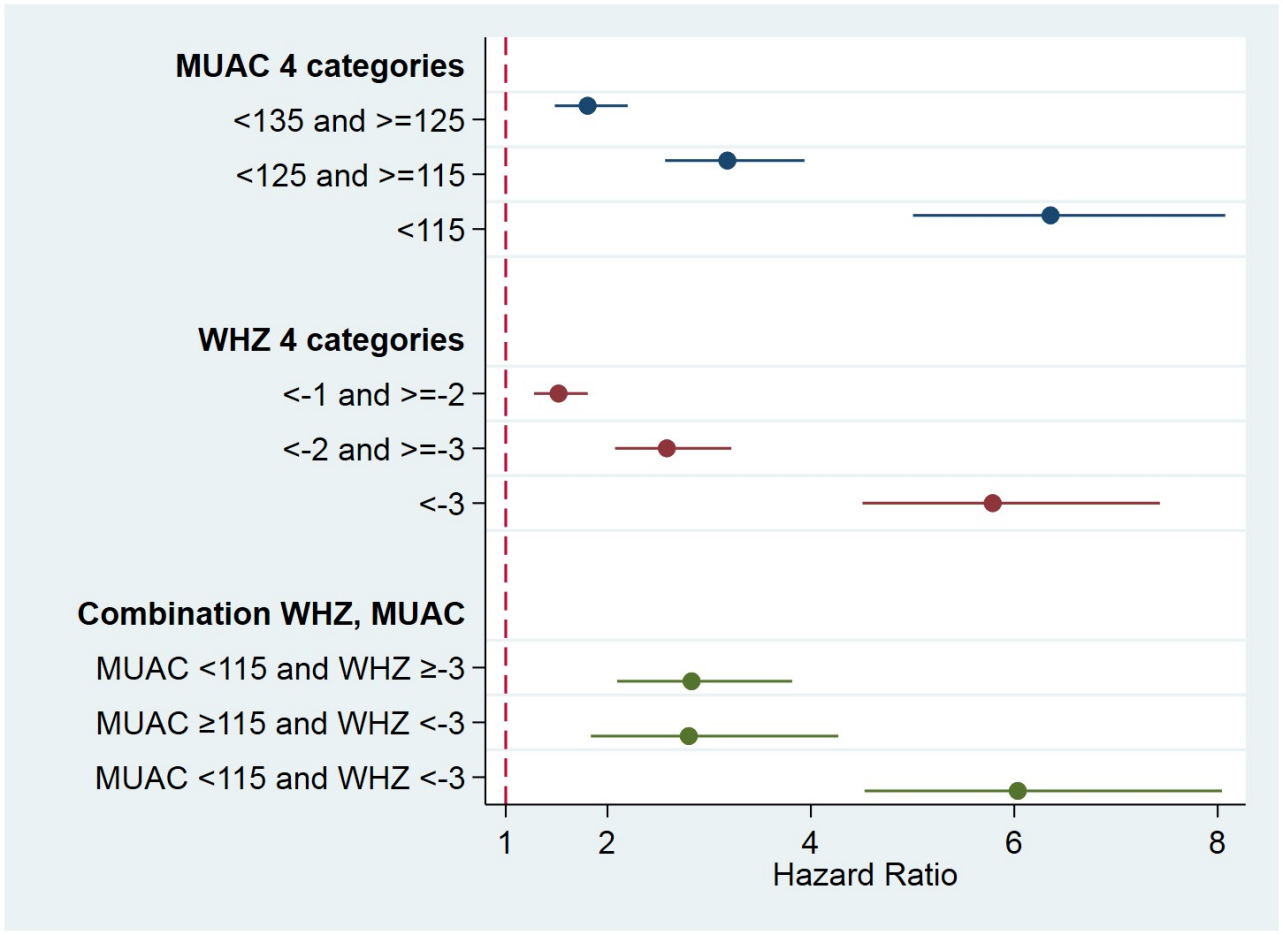
Hazard ratios with 95% CI resulting from Cox proportional hazard regression models for all measurements in children aged 6–59 months combined. The reference categories are ≥135 mm for MUAC; ≥-1 Z-scores for WHZ; and MUAC ≥115 and WHZ ≥-3 for the combination of indicators.

**Fig 3:**
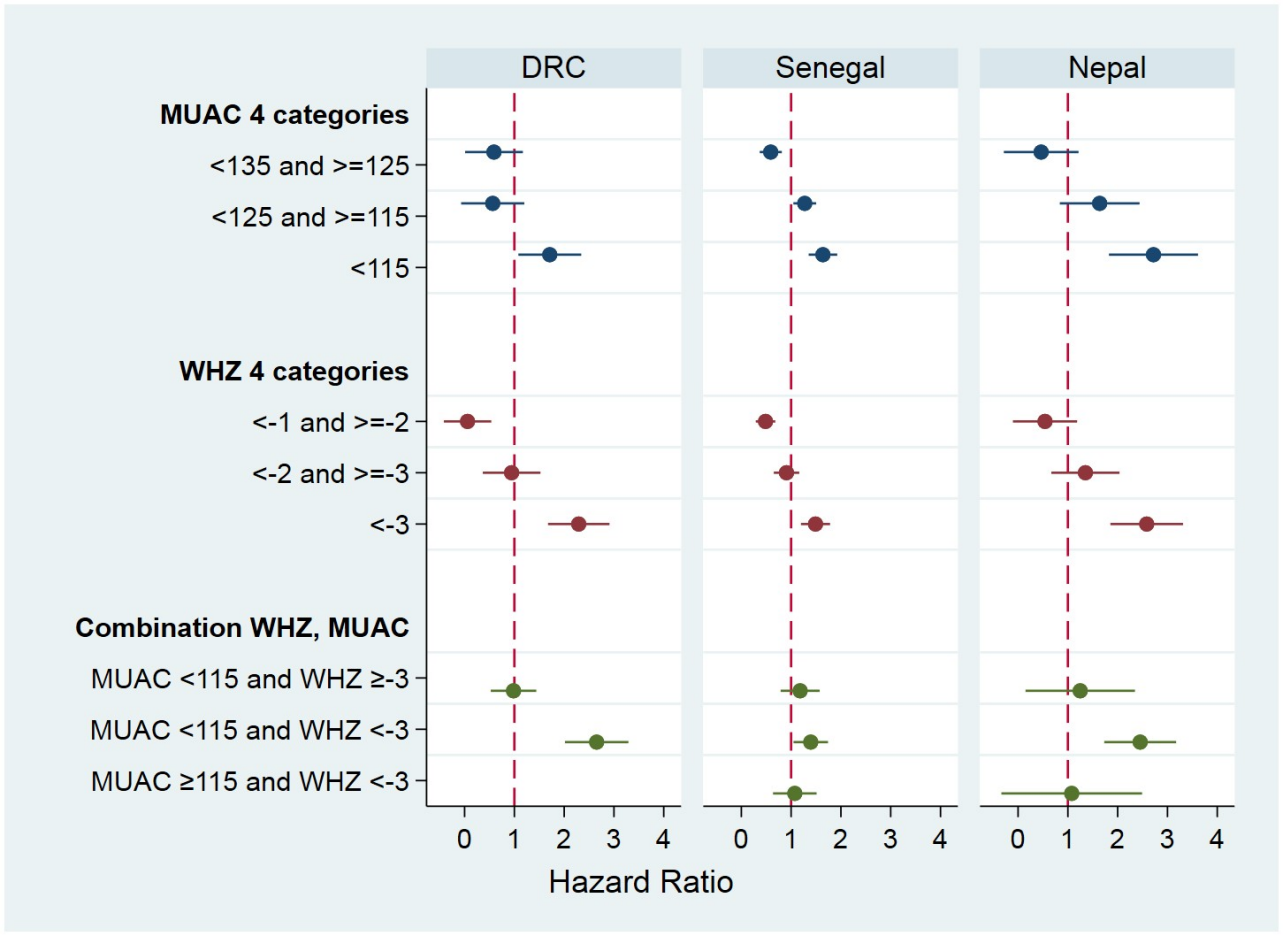
Hazard ratios with 95% CI resulting from Cox proportional hazard regression models separately for each of the three original studies. The reference categories are ≥135 mm for MUAC; ≥-1 Z-scores for WHZ; and MUAC ≥115 and WHZ ≥-3 for the combination of indicators.

Table 3 presents the number of children in the SAM categories that died before the next visit to the household. The Cox proportional hazards regression models show that the mortality hazard (hazard ratio; HR) increased exponentially as the anthropometric deficit increased for all anthropometric indices (Fig 2). The mortality hazard was elevated for children identified as SAM by MUAC <115mm (HR 3.96) and those identified by WHZ <-3 (HR 4.53) as compared to the reference category (MUAC ≥115 and WHZ ≥-3 respectively); the 95% CIs in these two categories were largely overlapping (Table 3). Those with only a single deficit had an almost 3 times higher mortality hazard than those with neither criterion below the cut-off. Having both indices under the cut-off for SAM, children had a statistically significant higher hazard of death (HR 6.12) than if only one index was under the cut-off (p<0.001 compared to MUAC-only and p = 0.002 compared to WHZ-only category). The case fatality rates (CFR) give similar results (Table 3), except that the CFR was higher for children who had SAM by WHZ (independent of MUAC) than those who had SAM by the MUAC criterion with a rate ratio of 1.46 (95%CI 1.10, 1.95). The case fatality rates reported in Table 3 translate into 1.31 deaths/10,000 children/day for the reference category of “normal” children (i.e. MUAC ≥135mm and WHZ ≥-1). For the other categories, values using this unit (deaths/10,000 children /day) are 5.08 (MUAC <115mm), 7.45 (WHZ <-3), 3.55 (MUAC-only), 4.13 (WHZ-only), and 9.60 (both MUAC and WHZ). Results stratified according to study (DRC, Senegal and Nepal) are presented in Fig 3; details can be found in the supporting information S2 Table.

**Table 3:** Case fatality rate (CFR)^a^ and hazard ratio (HR) resulting from Cox proportional hazard regression models^b^ for all measurements in children aged 6–59 months combined.

	Person time^c^	Deaths (N)	CFR	95% CI	Hazard Ratio	95%CI
**MUAC, mm** *[4 categories]*						
≥135	79,330	313	0.39	0.35, 0.44	Ref	
<135 and ≥125	37,690	174	0.46	0.40, 0,53	1.81	1.48, 2.20
<125 and ≥115	19,216	145	0.75	0.64, 0.88	3.19	2.57, 3.95
<115	7,275	117	1.61	1.34, 1.93	6.44	5.07, 8.18
**WHZ** *[4 categories]*						
≥-1	94,584	382	0.40	0.36, 0.44	Ref	
<-1 and ≥-2	33,779	184	0.54	0.47, 0.62	1.52	1.28, 1.81
<-2 and ≥-3	11,704	105	0.90	0.74, 1.09	2.57	2.07, 3.21
<-3	3,444	78	2.26	1.81, 2.82	5.83	4.55, 7.47
**Severe acute malnutrition (SAM)**						
**MUAC, mm** *[2 categories]*						
MUAC ≥115	136,237	632	0.46	0.43, 0.50	Ref	
MUAC <115	7,275	117	1.61	1.34, 1.93	3.96	3.19, 4.91
**WHZ** *[2 categories]*						
WHZ ≥-3	140,068	671	0.48	0.45, 0.52	Ref	
WHZ <-3	3,444	78	2.26	1.81, 2.82	4.53	3.57, 5.73
**combination MUAC, WHZ and both with non-SAM children as reference**						
MUAC ≥115 and WHZ ≥-3	134,883	615	0.46	0.43, 0.50	Ref	
MUAC <115 and WHZ ≥-3	5,185	56	1.08	0.83, 1.40	2.84	2.10, 3.84
MUAC ≥115 and WHZ <-3	1,354	17	1.26	0.78, 2.03	2.77	1.82, 4.23
MUAC <115 and WHZ <-3	2,089	61	2.92	2.27, 3.75	6.12	4.60, 8.13
**combination MUAC, WHZ and both with normal children as reference**						
MUAC ≥135 and WHZ ≥-1	67,468	269	0.40	0.35, 0.45	Ref	
MUAC <115 and WHZ ≥-3	5,185	56	1.08	0.83, 1.40	4.06	2.83, 5.83
MUAC ≥115 and WHZ <-3	1,354	17	1.26	0.78, 2.03	3.69	2.38, 5.71
MUAC <115 and WHZ <-3	2,089	61	2.92	2.27, 3.75	8.32	6.11, 11.3

^a^ The date of death was ascertained at the end of the observation period which had a median length of 4 months (IQR 3–5 months); the CFR is expressed as number of death per 100 child-months

^b^ Cox PH bivariable models with child’s age as time scale, stratified on cohort to account for significant cohort differences. Models account for repeated measurements for each child

^c^ Time contributed measured as child-months

**Fig 4:**
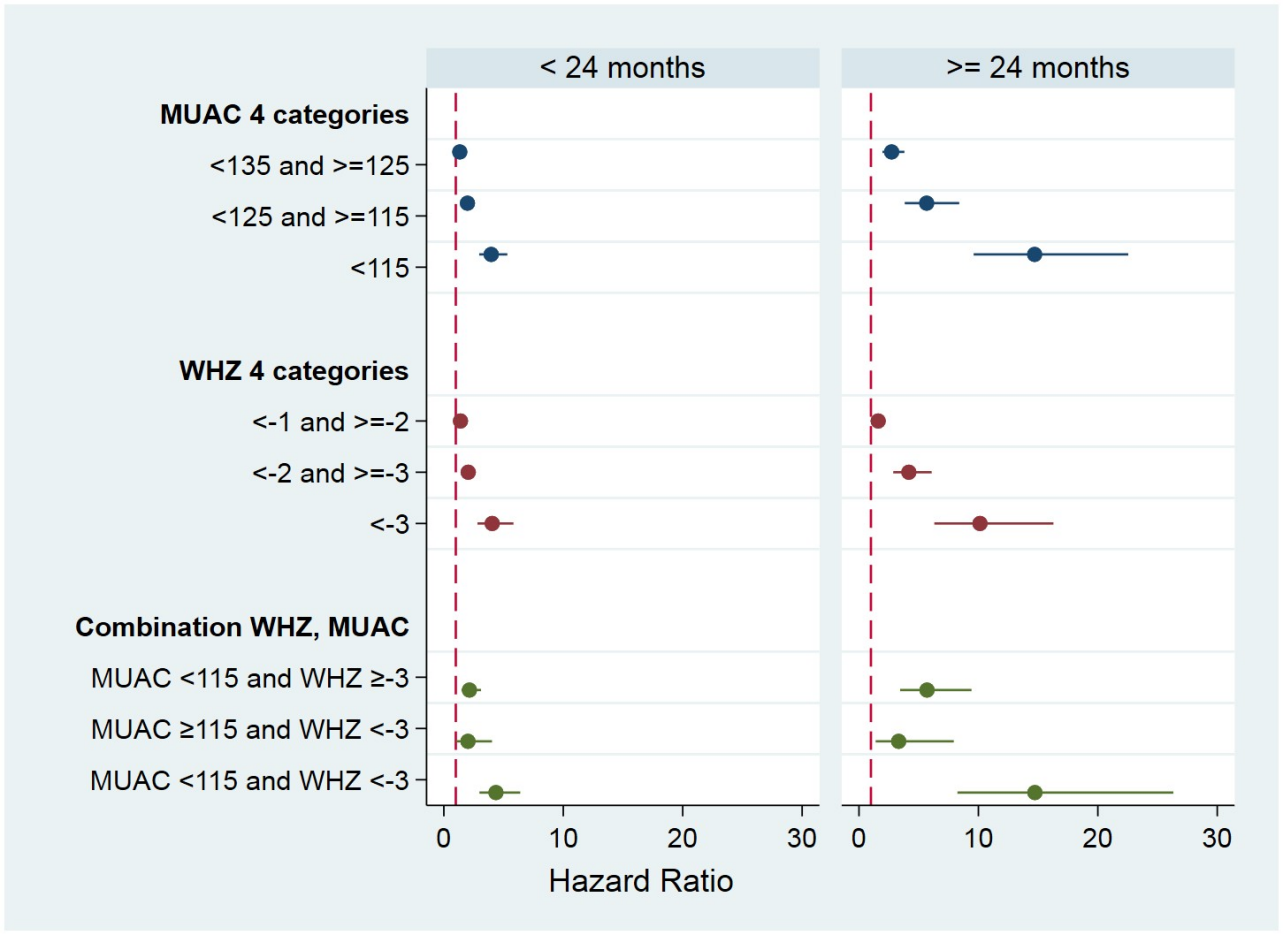
Hazard ratios with 95% CI resulting from Cox proportional hazard regression models according to age group. The reference categories are ≥135 mm for MUAC; ≥-1 Z-scores for WHZ; and MUAC ≥115 and WHZ ≥-3 for the combination of indicators.

We found a significant interaction of the anthropometric indices with age. A stratified analysis by age group is shown in Fig 4 (and supporting information S3 Table). The hazard ratios (HR) were greater for children ≥ 24 months compared to younger children for all exposure categories (the WHZ-only category for SAM was not significant). As expected the mortality rate of normal older children was about half that of the younger children; there was a slightly lower fall in mortality rate between the age groups in the mildly malnourished groups. However, the reduction in mortality with age did not occur with moderately malnourished children, and the mortality rate was substantially higher in the severely malnourished older children than younger children assessed with either WHZ or MUAC, and particularly in those with both deficits.

There was no significant interaction of anthropometric indices with stunting status (S4 Table). The mortality rates for stunted compared to non-stunted children indicate that in these cohorts the stunted children with a normal MUAC or WHZ was lower than for those who were not stunted. In children with a MUAC <115 mm only, the mortality rates for stunted children was higher than those without stunting. However, this was different for children with only a WHZ <-3, which if stunted had half the mortality rate of those who were of normal height for age. This appears to be a dominant effect because stunted children with SAM by both criteria had half the mortality rate of non-stunted children. However, for all categories, confidence intervals overlap.

The increasing mortality hazard in children with an increasing anthropometric deficit was also seen when the data were analyzed by cause of death for children that died primarily due to diarrheal diseases, respiratory diseases, or measles (S5 Table). The hazard of child death due to malaria was not associated with anthropometric status. Only 12 children were recorded as dying from traumatic incidents (accidents, sudden infant deaths, snakebite or food poisoning). A Cox regression model was not generated for trauma; the mean WHZ and the mean MUAC were not different for these children compared to the survivors (independent t-test).

## Discussion

We have described the mortality hazard associated with SAM by MUAC, WHZ and both criteria using WHO recommended standards for the same groups of children living in the community where therapeutic treatment was not available. In our analysis pooling data of over 15,000 children aged 6–59 months from three community-based cohorts, we find that the mortality risk increases exponentially with decreasing anthropometric status measured by both WHZ and by MUAC. Children identified as having SAM only by MUAC and only by WHZ had a similar mortality hazard (HR 4.06 and 3.69 respectively) and this hazard was even higher when both MUAC and WHZ were below the SAM cut-off (HR 8.32) compared to children within the normal range.

That low WHZ and low MUAC identify different children has been shown repeatedly [2–8]. In community surveys, only 16.5% of children had SAM by both deficits [9]. Only the children with dual deficits would be identified as SAM in both a MUAC-only and a WHZ-only program; if only one anthropometric measurement were to be made, those children with the alternative deficit would be excluded from treatment. Therefore, in order to determine the effect of using only a single anthropometric criterion to identify children with SAM, is it critical to examine the potential fate of children with single deficits, i.e. either MUAC <115mm or WHZ <-3Z without the other deficit. Children with single deficits had about the same mortality hazard when they have SAM by MUAC and SAM by WHZ alone. Our multi-country analysis of untreated children in the community thus confirms and gives similar results to the analysis of the mortality risks by diagnostic category of patients with SAM under treatment [18] as well as a systematic review and meta-analysis of studies comparing MUAC and WHZ mortality risks [19]. Thus, robust estimates of mortality risks derived from observations of cohorts of children in the absence of nutritional programs have now confirmed the findings mainly derived from patient data. The hazards ratio (HR) in our analysis was 6.44 for a severe deficit in MUAC (MUAC <115mm compared to ≥135mm) and 5.83 for WHZ <-3 (compared to ≥-1). A previous analysis pooling data of children aged 0–59 months from 10 studies, estimated the mortality hazard to be 11.6 times greater for a severe deficit in WHZ compared to a Z-score of -1 and above [20], but this study did not include the risks of a low MUAC in the same cohorts of children. The differences between the two analyses are the age range of the subjects eligible for analysis, the number of original studies included and the study contexts. However, as WHO recommendations for SAM management are targeted at children over 6 months [11], we deliberately excluded children less than 6 months from our analysis as these younger children have an inherently higher mortality rate (irrespective of their anthropometrical status) whose inclusion would bias results addressing children in the 6–59 month age range.

As nearly 84% of severely malnourished children had SAM by only one or the other deficit in the >1800 surveys analyzed by Grellety and Golden [9], failing to assess both criteria for SAM would result in large numbers of potential deaths being missed, and the prevalence of SAM would be underestimated. We agree with Wieringa et al. [28] that such a policy would be unethical. In regions where the caseload for WHZ is higher than MUAC, a MUAC-only programming would result in the majority of children with single deficits being excluded from treatment. An analysis of 733 small scale surveys from humanitarian crisis situations in 41 countries showed that the prevalence of wasting by WHZ was greater than that of wasting by MUAC in 74% of the included surveys [29]. The relative caseload not only varies dramatically between countries but also within countries such as Ethiopia [9], Somalia [30] and Cambodia [28]. This discrepancy within countries and ethnic groups has not, to our knowledge, been examined systematically elsewhere, has not been explained satisfactorily, and the effect of age, seasonality and other variables on the discrepancy has not been adequately assessed. However, the situation appears to be more complicated, as the population attributable fraction (a measure to indicate the proportion of deaths failed to prevent if a given indicator is not used) is dependent on the relative caseload and the hazard ratio. In our study population, 338 (13%) children would have been excluded from treatment under a MUAC-only programming; this is more pronounced in the Senegal cohort (29%) and the Nepal cohort (25%) (Fig 1), with a population attributable fraction (PAF) for death of 4.8% and 20.8% respectively. Nevertheless, each of the cohorts, from very different ecological and ethnic populations, give similar results in terms of the mortality hazards of children with SAM by MUAC and WHZ. Observed differences in risk estimates might point to varying contributions from underlying pathology, edematous malnutrition, or differences in average body constitution.

Those children fulfilling the criteria for SAM by both MUAC and WHZ had more than twice the mortality hazard of children with single deficits. This may be because these children are far below the SAM cut-off point for at least one of the criteria so that they then also become SAM by the alternative criterion; in effect, they have *very* severe acute malnutrition. Alternatively, it may indicate that these independent criteria not only identify different children, but also are indicative of different underlying metabolic changes. This is supported by studies in children showing different associations of MUAC or WHZ with body composition [31] and clinical features [6]. Thus, combining these pathologic processes would result in cumulating death risk associated with each type of malnutrition [7], and each indicator is not a proxy for the underlying pathological mechanisms of the other indicator.

### Age and mortality

In our study, hazard ratios for child mortality were higher for children older than two years for both MUAC and WHZ, and a combination of the two indices. Normal children’s MUAC increases steadily with age/height. As low MUAC is an absolute cut-off rather than a relative measurement adjusted for age or height, as a child gets older/taller the nutritional deficit with a MUAC <115mmm becomes steadily greater. Although a low MUAC is much more common in younger children, it carries less risk of death for these individuals than for older children; because with a progressive increase in the MUAC deficit with age, the risk of death increases exponentially. With the Cox analysis the reference mortality hazard is greater for the younger than the older children, so that the change in the denominator (i.e. the mortality hazard in the reference group) largely removes the effect of the inherently higher risk of death in younger children. However, these considerations do not account for the increased mortality risk in older children with a low WHZ. As well-nourished older children have a lower mortality rate than the younger children, the greatly increased hazard ratio, being a ratio to non-malnourished children, is in part due to the reduction in the denominator with age. High mortality in older compared to younger children has been noted elsewhere [32], but has not been frequently reported. Although it is expected that the mortality rate should be lower in older than younger children, this was not the case in either moderately or severely malnourished children with either a deficit in MUAC or WHZ (S3 Table). Therefore, the normal mortality reduction with age does not apply to malnourished children; this does not appear to have been described before. In this respect, it should be noted that older children are more likely to be SAM by WHZ than MUAC because WHZ is height, and therefore also age, adjusted. Although there are numerically fewer malnourished older than younger children, their high mortality risk makes this a particularly vulnerable group which has not been generally appreciated, except in famine situations [33, 34]. This raises the possibility that malnourished children older than 60 months may also experience a substantial risk of death; this should be a research priority. Our observations need to be confirmed elsewhere particularly in situations where the prevalence of acute malnutrition is high. Nevertheless, our finding challenges a key argument regularly put forward to justify the sole use of MUAC, which is that MUAC-only programs select younger children who are the most at-risk of death when they are malnourished.

### Stunting and mortality

Children with acute malnutrition who are also stunted are reported to have an increased mortality over those who are not stunted [35]. Because a stunted child is more likely to have a low absolute MUAC, this is another argument that has been advanced in favor of a MUAC-only program, although it should be noted that the contribution of an anthropometric deficit by MUAC <115mm was not considered in the study by McDonald et al. [35]. We did not find that stunting status modified the association between wasting and the mortality hazard in these three cohorts of children between 6 and 59 months. This is confirmed by Garenne et al. [36], who found that the interaction term between stunting and wasting did not add a considerable effect on the mortality risk in children aged 6–59 months in Senegal. Indeed, in our analysis, the relative mortality rates of stunted and non-stunted children gave some indication that stunted children had a lower mortality rate than non-stunted children which was particularly marked in the children with SAM by WHZ or both WHZ and MUAC. This finding is counter-intuitive. It appears that the relationships between malnutrition by MUAC, WHZ, stunting and age are more complicated than formally considered. One explanation could be that, because survival is suggested to be closely linked to muscle mass, and wasting is associated with an even greater decrease in muscle mass than stunting, the concomitant occurrence of stunting does not add substantially to the risk of mortality in those being wasted [37]. The present data should not be affected by a survivor bias; nevertheless, all such analyses might be subject to error because of confounding. The analysis by McDonald et al. [35] examined children from one week of age and followed the children for about one year (0.7 to 1.6 years). Their mortality data are likely to be dominated by infants, particularly small-for-gestational-age infants, who have a high mortality rate, especially in the first month of life. Our failure to find that concomitant stunting increased mortality may therefore be explained by the difference in the age range examined in the two studies. It would be useful for McDonald et al. [35] to re-examine their database to differentiate stunting and mortality in infants and children within different age ranges to see if this difference in findings can be reconciled. Nevertheless, if in children older than 6 months stunting does not augment the risk of death from SAM then this argument used by others to promote a MUAC-only program is without merit.

### SAM and cause-specific mortality

In our cause-specific analysis, deaths with a primary cause of diarrhea, respiratory diseases and measles were associated with anthropometric status with the largest effect seen with diarrheal disease. Children with respiratory disease had a marginally increased hazard of mortality, but this did not reach significance in any group; this finding was unexpected [20, 38–40], and may be related to the power of the analysis as there were relatively few children dying from respiratory disease. Children dying of measles with only mild deficits with either anthropometric index were not significantly different to those in the reference category, but with greater deficits, there was a significant increase in mortality. This is in accordance with other studies [20, 41, 42]. In the study of Olofin et al. [20] risk of death due to malaria could not be studied, as the sample size was insufficient; in our study, malaria deaths were not related to anthropometrical status based on 87 deaths. Previous studies on death due to malaria and anthropometric status are inconsistent [38, 42]. For malaria, especially in community-based studies, the diagnosis is unreliable because fever, one of the main symptoms of malarial disease, is unspecific. It is also not established if a low anthropometric status is a protective or a risk factor for malaria [43, 44].

Deaths that could not be assigned to a specific cause were related to anthropometric status indicating potential misclassification and the limitation of verbal autopsy to ascertain the cause of death. This might have influenced the estimates for the cause-specific analysis.

### Strength and limitations

A main strength of our study is that we were able to gather rare community-based cohort studies that included all required measures and assess them using the WHO recommended standards. These studies are more likely to have minimized the risk of a selection bias compared to health facility based studies. However, selection bias in the original studies cannot be excluded. Publications reporting the original methods state that in Senegal 88.5% and in Nepal 96% of all the children of eligible ages were enrolled into the studies. However, in the Nepal study, we received a sub-set of the original study with 6,112 of the 28,630 children selected. We do not have any information on what criteria the selection of this sub-set was based. In the DRC dataset, the proportion of eligible children included is unknown although it is reported that 16 out of 52 villages in the study area were randomly selected and all mothers and children in these villages were enrolled into the study [45].

The studies investigated are quite old and there may have been changes in public health provision and general nutritional status since they were conducted. In order to investigate whether this could be a problem with interpretation of the data we have compared the ratios of SAM and GAM (Global Acute Malnutrition) obtained from surveys of randomly selected children in the three countries with the data from the historical cohorts used in this analysis. The data are shown in Table 4. Each of the 3 historical cohorts differ from the corresponding modern community-based surveys in the proportion of SAM and GAM identified by WHZ, MUAC or both criteria; this indicates that the cohort data might not represent the current situation in these countries. Relative to the modern surveys, there is a dearth of children with SAM and GAM by WHZ only and an excess of children with SAM and GAM by MUAC only; there is also an excess of children satisfying both criteria in the Senegalese and Nepalese cohorts. One explanation could be, that in our data the average number of child months contributed is greater in the younger age groups (6–23 months of age) compared to the older age groups (24–59 months of age). Younger children are more likely to be detected by MUAC and this could contribute to the higher MUAC-only caseload than in the representative surveys. However, this effect is unlikely to account for the magnitude of the discrepancy. Another possibility is that children were more likely to be referred for treatment at the local hospital when presenting with a low WHZ due to a higher perception of risk by WHZ at the time these cohort studies were conducted; hospital referral may then have led to the children being excluded from further study participation. The ratio of SAM prevalence by diagnostic category in the DRC study seems somewhat unusual with a very low proportion of children with low WHZ in comparison to both the Senegal and Nepal study in this analysis, and other community-based studies [3, 4, 9]. Based on the original report, the occurrence of edema was low in the DRC study (on average 0.1% at each time point) [45]. Edema often appears acutely before death, and the incidence might not have been captured adequately in the 3-monthly surveys in the DRC study. Although we do not have data on edema for the Senegal and the Nepal cohort, this is not likely to explain the discrepancy between the cohorts and the surveys. When repeating the main Cox regression analysis excluding the DRC dataset, the estimates were similar and we would have arrived at the same conclusions (results not shown). However, the different proportions in these historical studies also points to a possible limitation of previous analyses on the same study population that have been used to advocate for MUAC-only programming. These are based on a higher sensitivity and specificity of MUAC to predict death using ROC curve comparisons [6, 12, 14, 46, 47]. In fact, differences in sensitivity and specificity for death between indicators greatly depend on difference in caseloads [48], which appear to be exceptionally in favor of MUAC in these historical surveys, as compared with recent representative cross-sectional surveys. Thus, if the study sample is not representative of the target population, the analysis can be misleading unless the mortality rates are related to the relative “population attributable mortality”. A high proportion of children with both MUAC and WHZ deficits, which are then incorporated into both arms of a comparison of their relative mortality rates can lead to the statistical error of mathematical coupling [18, 49, 50] which could even lead to reversal of the relative mortality rates (Simpson’s paradox) [51]; it is for that reason that we present the mortality data for those with only a low WHZ and a low MUAC separately from those with both deficits.

**Table 4:** Comparison of the proportions of children with Global Acute Malnutrition (GAM) and Severe Acute Malnutrition (SAM) obtained from modern randomly selected community surveys and from the historical cohorts analyzed in the present study.

		subjects	MUAC-only^a^	WHZ-only^b^	Both criteria
		#	%	%	%
		**SAM**			
**Senegal**	**Surveys**^c^	534	6.7	86.0	7.3
	**Cohort**	388	36.3	28.9	34.8
**DRC**	**Surveys**^c^	4,683	55.4	34.8	9.8
	**Cohort**	1,495	90.6	2.3	7.2
**Nepal**	**Surveys**^c^	238	36.6	43.3	20.2
	**Cohort**	772	38.2	24.9	36.9
		**GAM**			
**Senegal**	**Surveys**^c^	3,648	6.8	78.4	14.8
	**Cohort**	1,510	31.5	27.5	41.1
**DRC**	**Surveys**^c^	23,416	42.7	32.6	24.6
	**Cohort**	5,284	84.4	1.9	13.7
**Nepal**	**Surveys**^c^	1,082	26.6	39.4	34.0
	**Cohort**	3,225	28.2	28.5	43.3

^a^ children with a MUAC<115mm for SAM and MUAC <125mm for GAM, but a WHZ ≥-3 and-2 respectively

^b^ children with a WHZ <-3 for SAM and WHZ<-2 for GAM, but a MUAC≥115 or 125mm respectively

^c^ data is taken from Grellety and Golden [9]

Despite pooling individual-level data for analysis, the sample size in some categories for sub-analysis was low, reducing the power to draw clear conclusions on some of the (secondary) objectives. In the included cohorts, the death rate was lower than expected. Even in the absence of nutritional programs, children found severely ill were referred to the next health station (DRC, Nepal) or strongly advised to visit the collaborating hospital (Senegal). This could have biased our estimates of death rates.

Another limitation refers to the question of whether the datasets we received were thoroughly cleaned. When we checked for consistency at subsequent time points, we found that many anthropometric measurements were not plausible based on criteria defined prior to our analysis (see S1 Table). The surprising number of data-points that we had to eliminate during the initial cleaning calls into question the quality control of the original measurements. At the time these studies were conducted it was considered that increasing sample size would counteract the effect of random measurement error; we now recognize this to be an incorrect assumption [52]. Nevertheless, estimates of mortality hazard (HR) did not change substantially when we applied quite stringent cleaning criteria (S1 Table) and therefore we are confident that this would not have affected the conclusions presented in this analysis.

## Conclusions

In the light of the ongoing debate about the increase in use of MUAC as the sole indicator for defining severe acute malnutrition, we contribute a comprehensive analysis of the mortality hazard for the different anthropometric indicators in children in the community rather than in facilities or programmatic settings. We found that children identified as having SAM by only MUAC <115mm had a similar hazard of death as those identified with only a WHZ <-3. If MUAC was used as the sole indicator, wasted children by WHZ with a similarly increased risk of death would be excluded from diagnosis. We would expect this proportion to be higher in contexts where there is a high SAM caseload by WHZ criteria, and probably in severe crisis situations, where WHZ-only diagnosis tends to increase in prevalence more than MUAC-only [29]. While it is clear that there is a need for simple tools for case finding, especially in emergency settings, with the extent of high-risk children being missed for treatment, we think it would be unethical not to use WHZ whenever possible.

## Supporting information

S1 Table Results from Cox proportional hazard regression models for all measurements in children combined using the dataset with additional cleaning criteria. (DOCX)

S2 Table Hazard ratios (HR) resulting from Cox proportional hazard regression models separately for each of the three original studies. (DOCX)

S3 Table Case fatality rate (CFR) and hazard ratio (HR) resulting from Cox proportional hazard regression models according to age group. (DOCX)

S4 Table Case fatality rate (CFR) and hazard ratio (HR) resulting from Cox proportional hazard regression models according to stunting status. (DOCX)

S5 Table Results from Cox proportional hazard regression models according to selected causes of death. (DOCX)
