# Supplementary material for: Severe acute malnutrition and mortality in children in the community: Comparison of indicators in a multi-country pooled analysis
Source: PLoS One. 2019 Aug 6;14(8):e0219745. doi: 10.1371/journal.pone.0219745 (PMC6684062; doi:10.1371/journal.pone.0219745)
Supplement: S1 Table — (DOCX) [file pone.0219745.s001.docx]

**S1 Table. Results from Cox proportional hazard regression models^a^ for all measurements in children combined using the dataset with additional cleaning criteria ^b^**

|  | Person time^c^ | Deaths (N) | Hazard Ratio* | 95%CI |
| --- | --- | --- | --- | --- |
| MUAC, mm *[4 categories]* |  |  |  |  |
| ≥135 | 77,685 | 295 | Ref |  |
| <135 and ≥125 | 36,414 | 162 | 1.81 | 1.47, 2.22 |
| <125 and ≥115 | 17,393 | 134 | 3.24 | 2.59, 4.06 |
| <115 | 5,578 | 99 | 6.44 | 4.98, 8.33 |
|  |  |  |  |  |
| WHZ *[4 categories]* |  |  |  |  |
| ≥-1 | 91,062 | 357 | Ref |  |
| <-1 and ≥-2 | 32,623 | 172 | 1.51 | 1.26, 1.80 |
| <-2 and ≥-3 | 10,727 | 90 | 2.40 | 1.90, 3.04 |
| <-3 | 2,660 | 71 | 6.35 | 4.90, 8.24 |
|  |  |  |  |  |
| Severe acute malnutrition (SAM) |  |  |  |  |
| MUAC, mm *[2 categories]* |  |  |  |  |
| MUAC ≥115 | 131,494 | 591 | Ref |  |
| MUAC <115 | 5,578 | 99 | 3.89 | 3.08, 4.92 |
|  |  |  |  |  |
| WHZ *[2 categories]* |  |  |  |  |
| WHZ ≥-3 | 134,413 | 619 | Ref |  |
| WHZ <-3 | 2,660 | 71 | 4.99 | 3.90, 6.39 |
|  |  |  |  |  |
| combination MUAC, WHZ |  |  |  |  |
| MUAC ≥115 and WHZ ≥-3 | 130,308 | 575 | Ref |  |
| MUAC <115 and WHZ ≥-3 | 4,104 | 44 | 2.46 | 1.75, 3.46 |
| MUAC ≥115 and WHZ <-3 | 1,185 | 16 | 2.95 | 1.29, 4.52 |
| MUAC <115 and WHZ <-3 | 1,474 | 55 | 6.88 | 5.09, 9.28 |

^a^ Cox PH bivariable models with child’s age as time scale, stratified on cohort to account for significant cohort differences. Models account for repeated measurements for each child

^b^ additional data cleaning criteria for adjacent measurements in individual children standardized to 3-month periods: decrease or >4cm gain in height; +/- more than 20% change in weight, +/- more than 2cm change in MUAC

^c^ Time contributed measured as child-month
