# Supplementary material for: Severe acute malnutrition and mortality in children in the community: Comparison of indicators in a multi-country pooled analysis
Source: PLoS One. 2019 Aug 6;14(8):e0219745. doi: 10.1371/journal.pone.0219745 (PMC6684062; doi:10.1371/journal.pone.0219745)
Supplement: S2 Table — (DOCX) [file pone.0219745.s002.docx]

**S2 Table. Hazard ratios (HR) resulting from Cox proportional hazard regression models** ^a^ **separately for each of the three original studies**

|  | **DRC** | | | | **Senegal** | | | | **Nepal** | | | |
| --- | --- | --- | --- | --- | --- | --- | --- | --- | --- | --- | --- | --- |
|  | Person time ^b^ | deaths | HR | 95%CI | Person time ^b^ | deaths | HR | 95%CI | Person time ^b^ | deaths | HR | 95%CI |
| **MUAC, mm** *[4 categories]* |  |  |  |  |  |  |  |  |  |  |  |  |
| ≥135 | 15,038 | 19 | Ref |  | 34,794 | 275 | Ref |  | 29,498 | 19 | Ref |  |
| <135 and ≥125 | 11,863 | 37 | 1.84 | 1.02, 3.28 | 9,789 | 121 | 1.80 | 1.44, 2.25 | 16,037 | 16 | 1.62 | 0.77, 3.43 |
| <125 and ≥115 | 8,765 | 29 | 1.80 | 0.95, 3.39 | 3,997 | 95 | 3.57 | 2.84, 4.49 | 6,454 | 21 | 5.28 | 2.38, 11.72 |
| <115 | 3,651 | 43 | 5.62 | 2.98, 10.60 | 1,253 | 46 | 5.15 | 3.86, 6.86 | 2,371 | 28 | 16.83 | 6.94, 40.80 |
| **WHZ** *[4 categories]* |  |  |  |  |  |  |  |  |  |  |  |  |
| ≥-1 | 30,574 | 79 | Ref |  | 33,771 | 284 | Ref |  | 30,239 | 19 | Ref |  |
| <-1 and ≥-2 | 6,629 | 21 | 1.07 | 0.67, 1.73 | 11,208 | 144 | 1.62 | 1.33, 1.97 | 15,942 | 19 | 1.71 | 0.90, 3.27 |
| <-2 and ≥-3 | 1,726 | 15 | 2.55 | 1.42, 4.57 | 3,817 | 69 | 2.47 | 1.91, 3.20 | 6,160 | 21 | 3.88 | 1.95, 7.69 |
| <-3 | 388 | 13 | 9.46 | 5.20, 17.18 | 1,037 | 40 | 4.43 | 3.31, 5.94 | 2,019 | 25 | 14.03 | 6.80, 28.92 |
| **SAM** |  |  |  |  |  |  |  |  |  |  |  |  |
| **MUAC, mm** *[2 categories]* |  |  |  |  |  |  |  |  |  |  |  |  |
| MUAC ≥115 | 35,667 | 85 | Ref |  | 48,581 | 491 | Ref |  | 51,989 | 56 | Ref |  |
| MUAC <115 | 3,651 | 43 | 3.52 | 2.33, 5.32 | 1,253 | 46 | 3.58 | 2.47, 4.67 | 2,371 | 28 | 8.08 | 4.09, 15.94 |
| **WHZ** *[2 categories]* |  |  |  |  |  |  |  |  |  |  |  |  |
| WHZ ≥-3 | 38,930 | 115 | Ref |  | 48,797 | 497 | Ref |  | 52,341 | 59 | Ref |  |
| WHZ <-3 | 388 | 13 | 8.49 | 4.75, 15.18 | 1,037 | 40 | 3.44 | 2.60, 4.54 | 2,019 | 25 | 8.08 | 4.36, 14.98 |
| **combination MUAC, WHZ** |  |  |  |  |  |  |  |  |  |  |  |  |
| MUAC ≥115 and WHZ ≥-3 | 35,561 | 85 | Ref |  | 48,131 | 477 | Ref |  | 51,190 | 53 | Ref |  |
| MUAC <115 and WHZ ≥-3 | 3,368 | 30 | 2.68 | 1.69, 4.25 | 665 | 20 | 3.74 | 2.51, 5.57 | 1,151 | 6 | 3.66 | 1.21, 11.02 |
| MUAC ≥115 and WHZ <-3 | 105 | 0 | - | - | 450 | 14 | 3.39 | 2.18, 5.26 | 799 | 3 | 2.83 | 0.68, 11.68 |
| MUAC <115 and WHZ <-3 | 282 | 13 | 13.47 | 7.31, 24.82 | 587 | 26 | 4.60 | 3.24, 6.53 | 1,220 | 22 | 12.76 | 6.23, 26.11 |

^a^ Cox PH bivariable models with child’s age as time scale, stratified on cohort to account for significant cohort differences. Models account for repeated measurements for each child

^b^ time contributed measured as child-months
