# Supplementary material for: Severe acute malnutrition and mortality in children in the community: Comparison of indicators in a multi-country pooled analysis
Source: PLoS One. 2019 Aug 6;14(8):e0219745. doi: 10.1371/journal.pone.0219745 (PMC6684062; doi:10.1371/journal.pone.0219745)
Supplement: S3 Table — (DOCX) [file pone.0219745.s003.docx]

**S3 Table. Case fatality rate (CFR)^a^ and hazard ratio (HR) resulting from Cox proportional hazard regression models ^b^ according to age group**

|  | <24 months | | | | | ≥24 months | | | | |  |  |
| --- | --- | --- | --- | --- | --- | --- | --- | --- | --- | --- | --- | --- |
|  | Person time ^c^ | deaths | CFR | HR | 95% CI | Person time^c^ | deaths | CFR | HR | 95% CI | CFR ratio | 95% CI |
| MUAC, mm |  |  |  |  |  |  |  |  |  |  |  |  |
| ≥135 | 27106 | 174 | 0.64 | **Ref** |  | 52224 | 139 | 0.27 | **Ref** |  | 0.41 | 0.33, 0.52 |
| <135 and ≥125 | 21819 | 114 | 0.52 | **1.33** | 1.06, 1.68 | 15871 | 60 | 0.38 | **2.73** | 1.96, 3.80 | 0.72 | 0.53, 0.99 |
| <125 and ≥115 | 14428 | 102 | 0.71 | **1.98** | 1.53, 2.56 | 4788 | 43 | 0.90 | **5.66** | 3.82, 8.38 | 1.27 | 0.89, 1.81 |
| <115 | 5850 | 77 | 1.32 | **3.96** | 2.96, 5.31 | 1424 | 40 | 2.81 | **15.3** | 10.03, 23.44 | 2.13 | 1.46, 3.13 |
| WHZ |  |  |  |  |  |  |  |  |  |  |  |  |
| ≥-1 | 39409 | 218 | 0.55 | **Ref** |  | 55174 | 164 | 0.30 | **Ref** |  | 0.54 | 0.44, 0.66 |
| <-1 and ≥-2 | 18672 | 126 | 0.67 | **1.39** | 1.12, 1.72 | 15107 | 58 | 0.38 | **1.61** | 1.19, 2.18 | 0.57 | 0.42, 0.78 |
| <-2 and ≥-3 | 8485 | 69 | 0.81 | **2.04** | 1.55, 2.68 | 3218 | 36 | 1.12 | **4.17** | 2.86, 6.09 | 1.38 | 0.92, 2.06 |
| <-3 | 2637 | 54 | 2.05 | **4.02** | 2.79, 5.78 | 807 | 24 | 2.97 | **10.6** | 3.65, 16.79 | 1.45 | 0.90, 2.35 |
| SAM |  |  |  |  |  |  |  |  |  |  |  |  |
| MUAC, mm |  |  |  |  |  |  |  |  |  |  |  |  |
| MUAC ≥115 | 63353 | 390 | 0.62 | **Ref** |  | 72883 | 242 | 0.33 | **Ref** |  | 0.54 | 0.46, 0.63 |
| MUAC <115 | 5850 | 77 | 1.32 | **3.00** | 2.29, 3.92 | 1424 | 40 | 2.81 | **8.17** | 5.20, 12.83 | 2.13 | 1.46, 3.13 |
| WHZ |  |  |  |  |  |  |  |  |  |  |  |  |
| WHZ ≥-3 | 66567 | 413 | 0.62 | **Ref** |  | 73501 | 258 | 0.35 | **Ref** |  | 0.57 | 0.48, 0.66 |
| WHZ <-3 | 2637 | 54 | 2.05 | **3.30** | 2.33, 4.67 | 807 | 24 | 2.97 | **7.85** | 4.94, 12.47 | 1.45 | 0.90, 2.35 |
| combination MUAC, WHZ |  |  |  |  |  |  |  |  |  |  |  |  |
| MUAC ≥115 / WHZ ≥-3 | 62446 | 378 | 0.61 | **Ref** |  | 72436 | 237 | 0.33 | **Ref** |  | 0.54 | 0.46, 0.64 |
| MUAC <115 / WHZ ≥-3 | 4120 | 35 | 0.85 | **2.14** | 1.47, 3.12 | 1064 | 21 | 1.97 | **5.38** | 3.53, 9.64 | 2.32 | 1.35, 3.99 |
| MUAC ≥115 / WHZ <-3 | 907 | 12 | 1.32 | **2.00** | 1.01, 4.00 | 447 | 5 | 1.12 | **3.26** | 1.36, 7.82 | 0.85 | 0.30, 2.40 |
| MUAC <115 / WHZ <-3 | 1730 | 42 | 2.43 | **4.33** | 2.96, 6.35 | 359 | 19 | 5.29 | **15.7** | 8.92, 27.69 | 2.18 | 1.27, 3.75 |

^a^ The date of death was ascertained at the end of the observation period which had a median length of 4 months (IQR 3-5 months; the CFR is expressed as number of death per 100 child-months

^b^ Cox PH bivariable models with child’s age as time scale, stratified on age category (below 24 months or 24 months and above), account for repeated measurements for each child and the study site

^c^ time contributed measured as child-months
