# Supplementary material for: Severe acute malnutrition and mortality in children in the community: Comparison of indicators in a multi-country pooled analysis
Source: PLoS One. 2019 Aug 6;14(8):e0219745. doi: 10.1371/journal.pone.0219745 (PMC6684062; doi:10.1371/journal.pone.0219745)
Supplement: S4 Table — (DOCX) [file pone.0219745.s004.docx]

**S4 Table. Case fatality rate (CFR)^a^ and hazard ratio (HR) resulting from Cox proportional hazard regression models ^b^ according to stunting status**

|  | Stunted (HAZ <-2 Z-scores) | | | | | Not Stunted (HAZ ≥-2 Z-scores) | | | | |  |  |
| --- | --- | --- | --- | --- | --- | --- | --- | --- | --- | --- | --- | --- |
|  | Person time ^c^ | deaths | Mortality rate | HR | 95% CI | Person time^c^ | deaths | Mortality rate | HR | 95% CI | Rate ratio | 95% CI |
| MUAC, mm |  |  |  |  |  |  |  |  |  |  |  |  |
| ≥135 | 33853 | 113 | 0.33 | **Ref** |  | 45476 | 200 | 0.44 | **Ref** |  | **0.76** | 0.60, 0.96 |
| <135 and ≥125 | 22157 | 102 | 0.46 | **1.95** | 1.46, 2.59 | 15532 | 72 | 0.46 | **1.48** | 1.12, 1.95 | **0.99** | 0.73, 1.34 |
| <125 and ≥115 | 12987 | 93 | 0.72 | **3.21** | 2.37, 4.36 | 6229 | 52 | 0.83 | **2.93** | 2.31, 4.03 | **0.86** | 0.61, 1.20 |
| <115 | 5864 | 100 | 1.71 | **7.18** | 5.20, 9.91 | 1410 | 17 | 1.21 | **4.71** | 2.73, 8.15 | **1.41** | 0.85, 2.37 |
| WHZ |  |  |  |  |  |  |  |  |  |  |  |  |
| ≥-1 | 45904 | 167 | 0.36 | **Ref** |  | 48679 | 215 | 0.44 | **Ref** |  | **0.82** | 0.67, 1.01 |
| <-1 and ≥-2 | 18900 | 104 | 0.55 | **1.65** | 1.29, 2.11 | 14878 | 80 | 0.54 | **1.31** | 1.02, 1.68 | **1.02** | 0.76, 1.37 |
| <-2 and ≥-3 | 7232 | 76 | 1.05 | **3.10** | 2.32, 4.14 | 4471 | 29 | 0.65 | **1.73** | 1.17, 2.56 | **1.62** | 1.06, 2.49 |
| <-3 | 2825 | 61 | 2.16 | **6.27** | 4.51, 8.73 | 619 | 17 | 2.75 | **5.13** | 3.30, 7.96 | **0.79** | 0.46, 1.35 |
| SAM |  |  |  |  |  |  |  |  |  |  |  |  |
| MUAC, mm |  |  |  |  |  |  |  |  |  |  |  |  |
| MUAC ≥115 | 68998 | 308 | 0.45 | **Ref** |  | 67239 | 324 | 0.48 | **Ref** |  | **0.93** | 0.79, 1.08 |
| MUAC <115 | 5864 | 100 | 1.71 | **3.88** | 3.00, 5.01 | 1410 | 17 | 1.21 | **3.29** | 1.92, 5.63 | **1.41** | 0.85, 2.37 |
| WHZ |  |  |  |  |  |  |  |  |  |  |  |  |
| WHZ ≥-3 | 72038 | 347 | 0.48 | **Ref** |  | 68030 | 324 | 0.48 | **Ref** |  | **1.01** | 0.87, 1.18 |
| WHZ <-3 | 2825 | 61 | 2.16 | **4.21** | 3.11, 5.69 | 619 | 17 | 2.75 | **4.57** | 2.96, 7.04 | **0.79** | 0.46, 1.35 |
| combination MUAC, WHZ |  |  |  |  |  |  |  |  |  |  |  |  |
| MUAC ≥115 / WHZ ≥-3 | 68091 | 299 | 0.44 | **Ref** |  | 66792 | 316 | 0.47 | **Ref** |  | **0.93** | 0.79, 1.09 |
| MUAC <115 / WHZ ≥-3 | 3947 | 48 | 1.22 | **2.87** | 2.05, 4.03 | 1238 | 8 | 0.65 | **2.00** | 0.93, 4.33 | **1.88** | 0.89, 3.98 |
| MUAC ≥115 / WHZ <-3 | 907 | 9 | 0.99 | **2.24** | 1.21, 4.15 | 447 | 8 | 1.79 | **3.41** | 1.97, 5.88 | **0.55** | 0.21, 1.44 |
| MUAC <115 / WHZ <-3 | 1917 | 52 | 2.71 | **5.76** | 4.07, 8.15 | 172 | 9 | 5.23 | **7.18** | 3.64, 14.18 | **0.52** | 0.26, 1.05 |

^a^ The date of death was ascertained at the end of the observation period which had a median length of 4 months (IQR 3-5 months); the CFR is expressed as number of death per 100 child-months

^b^ Cox PH bivariable models with child’s age as time scale, stratified on stunting status (below 2 Z-scores or 2 Z-scores and above), account for repeated measurements for each child and the study site

^c^ time contributed measured as child-months
