# Supplementary material for: Severe acute malnutrition and mortality in children in the community: Comparison of indicators in a multi-country pooled analysis
Source: PLoS One. 2019 Aug 6;14(8):e0219745. doi: 10.1371/journal.pone.0219745 (PMC6684062; doi:10.1371/journal.pone.0219745)
Supplement: S5 Table — (DOCX) [file pone.0219745.s005.docx]

**S5 Table. Results from Cox proportional hazard regression models ^a^ according to selected causes of death**

|  | **Diarrhea** | | **Respiratory disease** | | **Measles** | | **Malaria** | | **Unknown** | |
| --- | --- | --- | --- | --- | --- | --- | --- | --- | --- | --- |
|  | HR | (95%CI) | HR | 95% CI | HR | 95% CI | HR | 95% CI | HR | 95% CI |
| **MUAC, mm *[4 categories]*** |  |  |  |  |  |  |  |  |  |  |
| ≥135 | Ref |  | Ref |  | Ref |  | Ref |  | Ref |  |
| <135 and ≥125 | 2.27 | 1.56, 3.32 | 1.51 | 0.91, 2.50 | 1.18 | 0.58, 2.36 | 1.52 | 0.84, 2.73 | 1.63 | 1.06, 2.50 |
| <125 and ≥115 | 5.76 | 3.87, 8.57 | 1.15 | 0.54, 2.44 | 4.08 | 2.07, 8.05 | 1.67 | 0.82, 3.40 | 2.91 | 1.72, 4.91 |
| <115 | 10.58 | 6.87, 16.28 | 2.18 | 0.94, 5.00 | 5.43 | 2.18, 13.48 | 0.99 | 0.32, 3.01 | 5.26 | 2.96, 9.42 |
| **WHZ** *[4 categories]* |  |  |  |  |  |  |  |  |  |  |
| ≥-1 | Ref |  | Ref |  | Ref |  | Ref |  | Ref |  |
| <-1 and ≥-2 | 2.03 | 1.46, 2.81 | 1.21 | 0.74, 1.96 | 1.15 | 0.63, 2.11 | 1.12 | 0.65, 1.94 | 1.56 | 1.07, 2.27 |
| <-2 and ≥-3 | 3.86 | 2.62, 5.67 | 1.79 | 0.95, 3.38 | 2.72 | 1.32, 5.64 | 1.47 | 0.62, 3.45 | 1.58 | 0.87, 2.86 |
| <-3 | 7.18 | 4.50, 11.47 | 2.51 | 1.00, 6.30 | 4.46 | 1.77, 11.26 | 0.93 | 0.13, 6.78 | 5.51 | 2.99, 10.13 |
| **SAM** |  |  |  |  |  |  |  |  |  |  |
| **MUAC, mm** *[2 categories]* |  |  |  |  |  |  |  |  |  |  |
| MUAC ≥115 | Ref |  | Ref |  | Ref |  | Ref |  | Ref |  |
| MUAC <115 | 5.37 | 3.69, 7.80 | 1.81 | 0.84, 3.90 | 3.66 | 1.52, 8.78 | 0.70 | 0.25, 1.99 | 3.55 | 2.05, 6.15 |
| **WHZ** *[2 categories]* |  |  |  |  |  |  |  |  |  |  |
| WHZ ≥-3 | Ref |  | Ref |  | Ref |  | Ref |  | Ref |  |
| WHZ <-3 | 4.62 | 2.97, 7.18 | 2.19 | 0.89, 5.37 | 3.69 | 1.50, 9.08 | 0.88 | 0.12, 6.32 | 4.59 | 2.53, 8.33 |
| **combination MUAC, WHZ** |  |  |  |  |  |  |  |  |  |  |
| MUAC ≥115 and WHZ ≥-3 | Ref |  | Ref |  | Ref |  | Ref |  | Ref |  |
| MUAC <115 and WHZ ≥-3 | 4.48 | 2.72, 7.39 | 1.65 | 0.64, 4.24 | 4.70 | 1.66, 13.23 | 0.82 | 0.28, 2.36 | 1.37 | 0.49, 3.80 |
| MUAC ≥115 and WHZ <-3 | 2.42 | 0.95, 6.19 | 2.30 | 0.60, 8.80 | 5.77 | 1.84, 18.11 | 2.47 | 0.39, 16.68 | 0.98 | 0.14, 6.93 |
| MUAC <115 and WHZ <-3 | 6.65 | 4.06, 10.91 | 2.20 | 0.70, 6.89 | 2.71 | 0.67, 11.38 | - | - | 7.02 | 3.76, 13.11 |

^a^ Cox PH bivariable models with child’s age as time scale, stratified on cause of death, account for repeated measurements for each child and the study site. Number of children that died due to diarrheal diseases: 218, respiratory diseases: 102, measles: 75, malaria: 81 and unknown cause: 157
